# Malpositions of the Colon and Cœcum

**Published:** 1888-04

**Authors:** Thomas Walley

**Affiliations:** Principal Royal (Dick) Veterinary College, Edinburgh


THE JOURNAL

OF

COMPARATIVE MEDICINE AND SURGERY.

VOLUME IX. APRIL, 1888. No. 2.

### ORIGINAL COMMUNICATIONS.

### ART. VIII.—MALPOSITIONS OF THE COLON AND CŒCUM.

BY THOMAS WALLEY, M. R. C. V. S.
*Principal Royal (Dick) Veterinary College, Edinburgh.*

As already observed, malpositions are the most important of all the colic disorders, and it is a matter to which the attention of the profession in this country has only been directed for, comparatively, a very few years.

Some twenty-one years have elapsed since I realized the fact that many of the deaths from intestinal affections which were generally supposed to be due to enteritis were in reality not due to inflammation at all, but were the result of malpositions, of one form or other, of the colon—with strangulation, more or less complete, of its vessels.

Since the period mentioned, I have lost no opportunity of impressing what to me was a fact upon the minds of my professional brethren; not always, however, with success, for there is nothing more difficult to accomplish than to eradicate *old standing* notions from the minds of men. And, more importantly still, in the case of some of those whom one succeeds in convincing of their errors, they do not acknowledge the source from which such conversion has come, but publish their altered views as though they were entirely original. Occasionally, however, notable exceptions to this tendency are met with, and I have to acknowledge one such in the case of Mr. Fred. Smith, who has recently acknowledged (in a paper read by him at the last meeting of the National Veterinary Association) that his attention was first directed to the subject in question by the perusal of a paper which I read at a meeting of the Liverpool V. M. Association, in 1875.

A few months prior to the reading of that paper, Mr. Elam, of Liverpool, had publicly directed attention to the comparative frequency of twist of the colon and the possibility of successfully diagnosing and treating such cases.

The malpositions of the colon to which it is my present intention to refer, are

(a.) Simple Displacement.

(b.) Version.

(c.) Torsion,

And, as a result of these changes in position, Vascular Strangulation.

Simple Displacement consists simply of a lateral displacement of one of the curvatures of the colon and usually the pelvic, which is frequently thrown from its normal position in the left to the right lumbo-iliac region.

This displacement is not only of importance by virtue of the diminution which it brings about in the normal calibre of the lumen of the bowel itself, but by virtue also of the compression exerted on adjacent portions of intestine.

Version is of three forms :

(1.) *Lateral*, in which one of the flexures of the colon —most frequently the pelvic—is turned on its own axis, constituting partial torsion.

Here there is not only diminution by actual occlusion of the lumen of the intestine but compression of adjacent or sub-jacent portions of bowel also.

(2.) *Anteversion* is seen in the pelvic flexure which is frequently thrown forward—doubled upon itself—and is found on *post-mortem* examination, resting against the diaphragm ; and also in the right flexure, which is sometimes thrown forward upon itself and carries with it the cœcum, the apex of which, on *post-mortem* examination, is seen to be directed backwards instead of forwards.

(3.) *Retroversion* takes place in the diaphragmatic and asternal flexures which are doubled on themselves in a backward direction.

Torsion may be *partial* as in lateral version, or *complete*, *i. e.*, the bowel twisted round completely upon itself, and in some instances, twice. This condition may occur in any of the flexures.

Either of the above mentioned conditions may be *complicated* with such lesions as diaphragmatic, gastric or enteric rupture ; with mesenteric herniation of the small intestine ; with laceration of the omentum, the strands of which may become twisted round a knuckle of intestine and produce vascular strangulation or simple compression, with laceration of the abdominal parietes and, in pregnant mares, with uterine rupture and herniation of the bowel.

It need hardly be said that any one of the above mentioned complications materially adds to the gravity of the existing lesion.

Vascular Strangulation is only brought about when the compression exerted on any individual part of an intestine is sufficient to occlude the lumen of one or other of the sets of vessels, *i. e.*, the veins or arteries. In many instances of malposition, as in simple displacement and lateral version, no compression is exerted on the blood-vessels and, it is important to observe, no marked vascular lesions are to be found after death. Vascular Strangulation may be *venous* (partial) or *arterio-venous* (complete). At first sight it may be thought by some that there could scarcely be strangulation of one set of vessels without the other being involved. Such a conclusion, however, would be entirely erroneous as the amount of force necessary for the compression of a vein would be inoperative in the case of an artery ; and it may be noted here that while compression of the veins alone, or of both arteries and veins, ultimately brings about similar organic lesions, or at least lesions differing only in degree, yet there is a material difference in the train of symptoms presented during life and also in the length of time required to produce death.

Predisposing Causes are the anatomical characters and relations of the gut itself; manner of feeding; class of horse and the presence of one or more calculi in the intestine; or the growth of a tumor or tumors in connection with the intestinal wall. The first of these predisposing causes has already been alluded to; by manner of feeding, I mean any method likely to induce attacks of colic or indigestion and tympany, *e. g.*, irregular feeding and feed ng on indigestible or irritant foods. Calculi and abnormal growths are only operative in this direction in so far as that they, by their weight, favor the displacement of a knuckle of intestine when actual causes are brought into play.

That improper feeding has largely to do with the production of such conditions as these under consideration must be patent to all careful observers. To most it would probably appear that over-gorging would be the most likely condition to bring about malposition, but, except in the fact that it is often the cause of colic, it is less frequently a predisposing cause than is a comparative state of emptiness of the bowels.

As illustrative of this remark I may cite the case of a stud of horses belonging to one of my clients. For a considerable period deaths occurred with comparative frequency as a result of one form or other of malposition of the colon. After a considerable amount of enquiry the cause of this was traced to the system of feeding; it was found that the horses received the first supply of food at 4 A. M., they left the stables at from five to six o'clock and each carter took out with him his supply of food for the day in the shape of a liberal allowance of corn, with a very limited admixture of cut hay. The food so taken out was intended to serve for the mid-day meal, but if, as it sometimes happened, the services of the horses were not required through the morning the carters allowed them to eat the full supply of food for the day before the clock had struck the hour of ten; consequently it frequently came about that they received no more food until 6, 7 and sometimes 9 P. M. This system undoubtedly produced indigestion and unsuspected spasm of the bowels, especially in cases where the horses were allowed by the carters to imbibe an inordinate quantity of water during the afternoon or evening, the result being that the horses were frequently found showing symptoms of serious bowel disorder late at night or early on the following morning.

After full enquiry I advised a complete change in the system of feeding and this was followed by marked diminution in the number of cases of intestinal lesions.

In another and larger stud in which the management was of the most exact character the constant occurrence of such cases was for sometime a puzzle which I could not unravel; it received its solution in the discovery of the fact that the carters took advantage of every opportunity afforded them of appropriating for their horses everything in the shape of food and this, as it often happened, consisted of different kinds of cake and of brewer's draff. Strict orders were issued to the carters to discontinue this practice and at the same time a watch was kept over them by responsible officials with the result that important bowel complications became of comparative rarity in the stud.

For a long period I have held the opinion that violent spasmodic contraction of the bowels was sufficient in itself to produce malpositions and even twists, and this opinion was strengthened by observing that in cases of rabies in the dog—of the raging form—multiple intussusceptions of the bowels were discovered on post mortem examination; while in several cases of rheumatic cramps in the pig I had seen the rectum violently and intermittently extruded (prolapsed) and retracted.*

My attention was first directed to the probability of

*Furthermore, I detected, many years ago, the existence of *rotary torsion* in the bowels of a newly born monstrous calf; this could only have been produced by spasmodic contractions in the muscular coat of the bowel itself—the mesentery forming the axis upon which the rotation had taken place.

tympany acting as an actual cause of colic malposition by noting the effects of tympanitic distension on a knuckle of bowel when the abdomen was laid open in the making of *post-mortem* examinations.

But, while allowing sufficient latitude for the operation of the aforementioned causes in the production of bowel displacements, I must, nevertheless, emphatically state that in my opinion the vast majority of these cases are due to purely physical causes, *e. g.*, to horses rolling over in the agonies of colic or for their own pleasure ; to horses falling over embankments, or tripping up in their endeavors to start a load or to keep their feet on slippery roads in frosty weather. I have seen as many as three cases of torsion in one week during the continuance of treacherous weather in winter, and in each case the history has been that the horse had several times during the day, lost his footing on the slippery pavement of the streets ; and on one occasion not only was colic torsion produced by the violent attempts of a horse to regain his equilibrium after slipping up in the road, but the muscles of the abdomen and the adductor muscles of the thigh were found after death to be extensively lacerated, and one of the colic flexures was ruptured also.

In violent falls, or where animals are thrown over embankments, complications—(viz.: rupture of the diaphragm of the stomach or of the bowel) are more directly operative in the production of death than is the associated colic displacement.

The premonitory indications of the existence of grave colic lesions such as those under consideration are, in the vast majority of cases, neither very pronounced nor violent. I am perfectly satisfied that in many instances a displacement or twist exists for hours before any sign of pain is evinced ; the history of numbers of such cases being that the animal had been noticed a little dull during the day, had eaten the night's feed, but had been found about 9 or 10 P.M., showing unpleasant symptoms and before 6 A.M., dissolution has resulted. At other times, the driver of the horse will, when questioned, acknowledge "that he had observed symptoms of colic through the day but it had passed off and he did not think it necessary to mention it."

Frequently, the practitioner attends a horse suffering as he believes from colic for a period of several hours before any symptom is presented that is at all calculated to arouse suspicion as to the existence of any important lesion. From all this, I long since arrived at the conclusion that grave forms of malposition of the colon may exist, unsuspectedly, for hours, and that the development of diagnostic symptoms is coincident with the completion of vascular strangulation.

Symptoms of vascular strangulation are most pronounced when the venous circulation only is arrested.

After a period of several hours, during which the symtoms of colic only have been observed, the pulse becomes weaker and somewhat irregular, the breathing more accelerated and the countenance anxious ; it will be found that the posterior bowels have been rapidly emptied of their contents and the skin is (usually) bathed in a clammy perspiration ; but, more importantly than all this, it will be found that on attempts being made to "raise" the jugular vein for the purposes of venesection the effort is futile, or if the vein is distended sufficiently to enable the practitioner to open it, a very small quantity only of blood can be withdrawn and that is of a very dark color and plastic. The symptoms of colic are, as a rule, by no means violent, and indeed the animal often presents symptoms more closely allied to those of internal hemorrhage than colic. In some instances, however, enteric pain is manifested by marked violence prior to death.

In excessive strangulation of arteries and veins the symptoms are marked by extreme violence ; from the moment strangulation is accomplished the pain is agonizing and continuous, the animal dashes about utterly regardless of consequences, often clenches the teeth, sometimes neighs wildly and is usually dangerous to anybody who attempts to go about him. The pulse is strong, full and rapid, and blood flows freely and forcibly if the jugular vein is opened. The body is bedewed with perspiration, the mucous membranes highly injected, and, as in the case of venous strangulation, there is non-passage of fæces after the posterior bowel has emptied itself; violent straining and groaning is induced by the introduction of the hand into the rectum for the purpose of exploration. The countenance assumes a Hippocratic expression prior to death. Venesection, even to the production of syncope, does not materially moderate the symptoms in these cases.

In partial compression or strangulation of the vessels the symptoms are much less violent than in either of the preceding conditions; they are often intermittent. There may be a moderate degree of enteric pain, sometimes perspiration, the pulse is for a long time, but little disturbed, afterwards it becomes rapid and weak and the respiration hurried; the temperature of the rectum is not materially increased nor is the color of the mucous membranes greatly heightened; the animal may eat at intervals and there is little or no anxiety of the countenance until a short time prior to dissolution.

Death seldom takes place in a less period than two or three days and I have known instances of prolongation of life for from five to seven days. As in the two former cases, evidence of mechanical occlusion is present in the form of non-passage or retention of fæces.

In reference to the *cause of death* in the three classes of cases described it may be observed that in *venous strangulation* it is due practically to *hemorrhage and effusion;* in *arterio-venous strangulation* it is due to *nervo-muscular exhaustion;* in *partial strangulation* to *systemic and nervous asthenia.*

M. Bouley (*Receuil de Medicine Veterinaire*) has expressed the opinion that in cases of vascular strangulation, death is due to absorption of septic matter from the intestines. I cannot endorse this opinion, as in the first two classes of cases noted, death takes place too rapidly for it to occur, and as the venous and lymphatic circulation of the involved intestine are practically segregated—it is improbable that absorption would take place through the tissues of contiguous bowels. In the third class of cases there is not usually sufficient post-mortem evidence of decomposition of the contents of the intestines or of liberation of poisonous gases to account for death by septicæmia or toxicœmia. Other evidence of the existence of the conditions described is to be found in the behavior of the animal, by rectal exploration and, in some of the third class of cases, in a symptom of gastric origin. In reference to the behavior of the animal it may indicate nothing more than bowel derangement; frequently, however, it will be seen that there is a great tendency to back and to rest the quarters on some prominent fixed object, such as a rack or manger; the hind legs are sometimes crossed, one in front of the other; the animal looks wistfully back at some particular part of the abdomen, sometimes assumes a kneeling, but, more largely a sitting posture; walks uneasily about and oftentimes circumperambulates the box for many minutes or for hours. He may lie down and roll on his back; may yawn or sigh, or may paw, more or less continuously, with one or both fore feet. None of these symptoms are necessarily characteristic, they may be concomitant of any bowel lesion of a mechanical nature.

By rectal exploration two important conditions may be detected (a) the presence in the pelvic cavity or on one side of the lumbar region of a resilient tumor, the fundus of which is presented *toward* the hand of the explorer. This tumor is produced by the decomposition of the contents of the involved knuckle of intestine and as a result of the liberation of gas; it is permanent and if displaced by pressure quickly returns. Mr. Elam has also particularly noticed the presence of this tumor and I have frequently directed the attention of my assistants and students to it in cases where *post-mortem* examination has proved the correctness of the diagnosis. I do not for a moment assert that this tumor is never found in any other conditions than those mentioned—it may be produced by the temporary occlusion of the lumen of a knuckle of intestine by the pressure of the mass of egesta in a contiguous portion of bowel and it is sometimes present in cases of impacted calculus; in the former case, however, the tumor disappears immediately, the bowels react. (b) On passing the hand into the rectum I have frequently detected the tense twisted condition of the muscucular bands of the colon, and in several instances have been thereby enabled to determine with accuracy the direction of the twist or displacement.

The gastric symptom of which I have spoken, consists in the usual indications of gastric tympany, and it is only seen when one of the flexures of the large colon is thrown across the duodenum or one of the small intestines thus preventing the onward passage of the contents of the stomach. The ingesta ferments, gas is liberated and persistent eructation results ; the stomach being usually found after death enormously distended with gas and fluids. The symptoms of gastric tympany usually make their appearance in these cases several hours subsequently to the relief of the primary colic.

*Treatment of Malpositions of the Colon.*—Before discussing this matter, one question may be considered, *i. e., the possibility of spontaneous reduction of such lesions.* Personally, I am more than convinced that such reduction is occasionally effected ; firstly, by a reversal of the spasm which gave rise to the condition, and, secondly, by the animal rolling ; or, in cases of anteversion, by the assumption of the sitting posture. I have, on several occasions, watched anxiously, and for hours together, cases of this kind in which all the symptoms pointed to the existence of one or other form of malposition. I have left my patient at a given time with the certainty in my own mind that he had only a short time to live and have returned in a few hours to find him relieved of all the distressing symptoms and in a fair way to recovery, and in each case the answer given by the attendant to my query as to when the patient's condition altered has been in some such words as, "Oh ! he got relief after he had suffered a violent attack of pain and had been rolling and tumbling about in the box, and after rising he 'broke wind' and his bowels began to rumble."

Such, I repeat, has been the kind of expression used by attendants on patients—under the circumstances mentioned—and after the lapse of a short time the bowels have re-acted and the animals have eventually made a good recovery.

While, in most instances of this kind, little benefit can be expected from the adoption of ordinary treatment all methods calculated to relieve pain and prevent exhaustion should be resorted to. Venesection is warranted on several grounds ; as a sedative, to relieve pressure or tension in the circulation ; to moderate effusion and hemorrhage, and to prevent pulmonary congestion—a sequel of common occurrence in cases where the animal recovers or is relieved. Paracentesis intestinalis should always be performed when tympany is at all marked. Cathartics are not only useless, they are absolutely poisonous. Intestinal sufflation by tobacco smoke may be of service, on the contrary, it may be extremely hurtful ; and the same may be said in reference to the use of special stimulants such as physostigimine and pilocarpine. In each case their use may be warranted as a *dernier resort.* In one case of torsion of the colon in which I found it necessary to bleed and to have recourse to tapping, I subsequently determined upon administering a dose of physostigimine. Evidence of its special action was quickly forthcoming, and after a short period of great suffering relief was afforded, a large quantity of gas was expelled *per anum*, the bowels re-acted, and the horse recovered.

In contemplating serious cases of this kind and realizing the inutility of ordinary methods of treatment, two courses of proceedure have, on various occasións, suggested themselves to me, viz.: to perform laparotomy and to reduce the mechanical lesion by passing the hand into the abdomen ; or, to reduce the twist by version of the body. The latter plan was suggested to my mind by the success attending its application in torsion of the uterus. I have put the plan into operation on several occasions, but in only one instance was indubitable evidence of its success forthcoming. The former plan I have not practiced owing to the opposition of owners and the uncertainty necessarily attending upon such an operation. I succeeded on one occasion in gaining the consent of the owner (I had tried turning of the body previously), but when I returned to the stable with the necessary instruments and hobbles, the owner had changed his mind and the patient was allowed to die.

If version of the body is determined upon, the patient must be cast or, if already recumbent, the legs must be firmly secured by the aid of ropes or hobbles. The direction of the twist having been previously determined by rectal exploration, the body of the horse must be rolled over in the *same direction* as that taken by the twist. The process will require to be repeated if the torsion is complete.

In the case I have referred to as being successful—the horse (a Clydesdale) had been ill for about forty-eight hours. The evidence as to the existence of twist was complete and the animal was almost in *extremis* when it was determined to practice version. Being much pressed for time I left the carrying out of the process in the hands of Mr. Wm. Reekie, M. R. C. V. S., who was at that time assisting me in practice ; the horse was rolled over twice and then released, he rose voluntarily, sighed heavily and immediately expelled a large volume of gas *per anum* and in a short time fæces were evacuated and the horse sought eagerly for food.

One question in the matter of general treatment may be briefly discussed, viz. : the advisability of allowing a horse to lie down and roll when evidence of the existence of some form of malposition is shown, and much might be said both for and against giving such liberty. Personally, I have often, as I have put it to myself, given the animal a chance of bringing about spontaneous reduction by allowing him to put himself in any position he felt inclined to assume.

The *post-mortem* evidences of malpositions of the colon having existed during life, are more complete and more satisfactory in those cases where the vascular strangulation has been pronounced, than in those in which it has been imperfect.

In the numerous autopsies I have made of such cases I have seldom failed to distinguish the evidence of fixation and vascular strangulation immediately the abdominal wall has been divided. The first thing that usually attracts attention is the existence of sero-sanguineous fluid in greater or lesser quantities in the abdomen and as the abdominal parietes are reflected, the incarcerated and tympanitic knuckle of intestine is extruded above the contiguous folds, its color depending on the amount of vascular strangulation; if the latter is very slight, the bowel may be of normal appearance, or, at most, may be moderately congested, or it may present evidence of circumscribed or limited extravasations of blood: if strangulation is more pronounced the existing lesions are characteristic.

They are confined to the involved portion of bowel and terminate abruptly at a given point, viz.: at the seat of compression. The bowel itself at this point is absolutely anœmic, and in cases of torsion the twisted fibres of the muscular wall of the intestine can be readily distinguished; more than this, if the torsion is reduced and the bowel released it tends to return to its original position.

The differences between the lesions presented in venous and arterio-venous strangulation are only differences in degree.

In *venous strangulation* the thickness of the intestine is extreme, often measuring one or one and a half to two inches through; the mucous membrane is extremely dark in color and friable; the small vessels markedly engorged, the interparietal connective tissue infiltrated extensively with serum which gives it on section a gelatinous appearance; the contents of the bowel are usually of a very dark color from the pouring out of sanguineous serum into the cavity of the intestine, and in some cases blood, imperfectly coagulated, is found mixed with the egesta; the colic veins are sur-charged with semi-coagulated blood of a very dark color and often of tarry consistence; the superficial lymphatics are distended with serum and the connective tissue surrounding the colic veins is œdematous.

In *arterio-venous strangulation* the conditions above described are exaggerated; the bowel is often gangrenous, the superficial mucosa dry, fissured, and of a very dark color—

sometimes green ; the mucous folds are very prominent and a gangrenous odor is evolved immediately the bowels is laid open. Intra-parietal and inter-parietal extravasation is marked and the muscular coat is very friable.

The conditions above noted are frequently attributed to enteritis, and, by some writers to septicæmia, but such misapprehensions are due in the first place to carelessness, in the second to prejudice, and in the third to ignorance—neither in enteritis nor in septicæmia do the lesions terminate abruptly nor is there the marked evidence of compression of the bowel that exists in the class of cases just considered.

